# Involvement of a putative acyltransferase gene in sporangium formation in *Actinoplanes missouriensis*

**DOI:** 10.1128/spectrum.04010-23

**Published:** 2024-03-19

**Authors:** Shixuan Hu, Satoshi Maeda, Takeaki Tezuka, Yasuo Ohnishi

**Affiliations:** 1 Department of Biotechnology, Graduate School of Agricultural and Life Sciences, The University of Tokyo, Bunkyo-ku, Tokyo, Japan; 2 Graduate School of Infection Control Sciences, Kitasato University, Minato-ku, Tokyo, Japan; 3 Collaborative Research Institute for Innovative Microbiology, The University of Tokyo, Bunkyo-ku, Tokyo, Japan; Institute of Microbiology, Chinese Academy of Sciences, Beijing, China

**Keywords:** *Actinoplanes missouriensis*, sporangium formation, acyltransferase, scanning electron microscopy, transmission electron microscopy

## Abstract

The actinomycete *Actinoplanes missouriensis* forms branched substrate mycelia during vegetative growth and produces terminal sporangia, each of which contains a few hundred spherical flagellated spores, from the substrate mycelia through short sporangiophores. Based on the observation that remodeling of membrane lipid composition is involved in the morphological development of *Streptomyces coelicolor* A3(2), we hypothesized that remodeling of membrane lipid composition is also involved in sporangium formation in *A. missouriensis*. Because some acyltransferases are presumably involved in the remodeling of membrane lipid composition, we disrupted each of the 22 genes annotated as encoding putative acyltransferases in the *A. missouriensis* genome and evaluated their effects on sporangium formation. The *atsA* (*AMIS_52390*) null mutant (Δ*atsA*) strain formed irregular sporangia of various sizes. Transmission electron microscopy revealed that some Δ*atsA* sporangiospores did not mature properly. Phase-contrast microscopy revealed that sporangium dehiscence did not proceed properly in the abnormally small sporangia of the Δ*atsA* strain, whereas apparently normal sporangia opened to release the spores. Consistently, the number of spores released from Δ*atsA* sporangia was lower than that released from wild-type sporangia. These phenotypic changes were recovered by introducing *atsA* with its own promoter into the Δ*atsA* strain. These results demonstrate that AtsA is required for normal sporangium formation in *A. missouriensis*, although the involvement of AtsA in the remodeling of membrane lipid composition is unlikely because AtsA is an acyltransferase_3 (AT3) protein, which is an integral membrane protein that usually catalyzes the acetylation of cell surface structures.

**IMPORTANCE:**

*Actinoplanes missouriensis* goes through a life cycle involving complex morphological development, including mycelial growth, sporangium formation and dehiscence, swimming as zoospores, and germination to mycelial growth. In this study, we carried out a comprehensive gene disruption experiment of putative acyltransferase genes to search for acyltransferases involved in the morphological differentiation of *A. missouriensis*. We revealed that a stand-alone acyltransferase_3 domain-containing protein, named AtsA, is required for normal sporangium formation. Although the molecular mechanism of AtsA in sporangium formation, as well as the enzymatic activity of AtsA, remains to be elucidated, the identification of a putative acyltransferase involved in sporangium formation is significant in the study of morphological development of *A. missouriensis*. This finding will contribute to our understanding of a complex system for producing sporangia, a rare multicellular organism in bacteria.

## INTRODUCTION

*Actinoplanes missouriensis* is a species of the genus *Actinoplanes* that is characterized by complex morphological development. During vegetative growth, it forms a branched substrate mycelium and produces globose or subglobose terminal sporangia that arise from the substrate mycelium via short sporangiophores (1). Inside a sporangium, a few hundred spherical spores are produced that are encased within a membranous substance called the spore sheath (2), and the space among the spores is filled with an intrasporangial matrix called the sporangium matrix (3). Spores are released from sporangia in response to water exposure via a process called sporangium dehiscence. Under laboratory conditions, sporangium dehiscence can be induced by pouring 25 mM NH_4_HCO_3_ solution onto sporangia formed on humic acid-trace element (HAT) agar and incubating the agar plate at room temperature for 1 h. Alternatively, sporangium dehiscence can be induced by suspending the sporangia harvested from the agar surface in 25 mM histidine solution, followed by incubation with rotation at room temperature for 1 h. After the release of spores from sporangia, the spore sheath is removed to generate zoospores, which can swim using flagella at high speed and exhibit chemotaxis to various types of compounds, such as sugars and amino acids (4, 5). Eventually, zoospores stop swimming, germinate, and grow into substrate mycelia. Due to its complex life cycle, *A. missouriensis* is of great interest for studying the molecular mechanisms of prokaryotic morphogenesis. When cultivated on HAT agar, *A. missouriensis* forms small sporangium-like structures within 2 or 3 days at 30°C. It then produces mature sporangia that can release spores after incubation of 5–7 days (6). The complete genome sequence of *A. missouriensis* has been determined previously (7).

In the model actinomycete *Streptomyces coelicolor* A3(2), which undergoes morphological development from vegetative mycelia to spores via aerial hyphae (8, 9), extensive variations in the membrane lipid composition have been observed during its developmental stages (10, 11). During sporulation, ornithine lipids (phosphorus-free polar lipids) accumulate, whereas phosphatidylethanolamine, one of the major lipids in the genus *Streptomyces*, is almost absent (10, 11). Furthermore, the inactivation of a gene (*SCO0921*) encoding an *N*-acyltransferase responsible for the synthesis of ornithine lipids caused precocious morphological development (10). In addition, the involvement of cardiolipins and phosphatidylinositols in the morphological development of *S. coelicolor* A3(2) has been suggested (12, 13). Considering these observations in *S. coelicolor* A3(2), we hypothesized that membrane lipid composition is also remodeled during sporangium formation in *A. missouriensis*, and it may be required for sporangium formation. Therefore, in this study, we focused on acyltransferases that may catalyze fatty acid transfer in lipid metabolism. Through a comprehensive gene disruption experiment on putative acyltransferase genes, we identified AtsA (AMIS_52390) as a putative acyltransferase required for normal sporangium formation in *A. missouriensis*.

## MATERIALS AND METHODS

### General methods

The bacterial strains, plasmid vectors, and media used in this study have been described previously (6, 14, 15). The primers used in this study are listed in Table S1. *A. missouriensis* was cultivated as previously described (3). Scanning electron microscopy (SEM) was performed using an S-4800 electron microscope (Hitachi, Tokyo, Japan) as previously described (16). Transmission electron microscopy (TEM) was performed using an H-7600 electron microscope (Hitachi) as described previously (3). Phase-contrast microscopy was performed using a BH-2 phase-contrast microscope (Olympus, Tokyo, Japan) as described previously (17). Free zoospores were quantified as previously described (18).

### Construction of gene deletion mutants

To construct the gene deletion mutants, the upstream and downstream regions (approximately 2 kbp each) of the target genes were amplified using PCR. The amplified DNA fragments were digested with restriction enzymes (see Table S1 for the enzymes used for each fragment) and cloned into pUC19 digested with the same restriction enzymes. The generated plasmids were sequenced to confirm the absence of PCR-derived errors. The cloned fragments were digested with appropriate restriction enzymes and cloned together into pK19mob*sacB* (19), whose kanamycin resistance gene had been replaced with the apramycin resistance gene (14). The generated plasmids were introduced into the wild-type *A. missouriensis* strain by conjugation, as described previously (20). Apramycin-resistant colonies resulting from single-crossover recombination were isolated. For each gene, one of them was cultivated in peptone-yeast extract-magnesium liquid broth at 30°C for 48 h, and the mycelia suspended in 0.75% NaCl solution were spread onto Czapek-Dox broth agar medium (BD, NJ, USA) containing extra sucrose (final concentration 5%). After incubation at 30°C for 4–5 days, sucrose-resistant colonies were inoculated onto yeast extract-beef extract-NZ amine-maltose monohydrate (YBNM) agar with or without apramycin to confirm that they were sensitive to apramycin. Apramycin-sensitive and sucrose-resistant colonies resulting from the second single-crossover recombination were isolated as candidates for gene deletion mutants. The deletion of each target gene was analyzed by PCR (data not shown).

### Construction of strains for gene complementation testing

A 1.7-kbp DNA fragment containing the promoter and coding sequences of *atsA* was amplified by PCR. The amplified fragment was digested with EcoRI and HindIII and cloned into pUC19 digested with the same restriction enzymes. The generated plasmid was sequenced to confirm that no PCR-derived errors were present. The cloned fragment was digested with EcoRI and HindIII and cloned into pTYM19-Apra (3, 21) digested with the same restriction enzymes. The generated plasmid was introduced into the *atsA* null mutant (Δ*atsA*) strain by conjugation as described previously (14). Plasmid pTYM19-Apra was also introduced into the wild-type and Δ*atsA* strains to generate the vector control strains. Apramycin-resistant colonies were isolated.

### Cappable-Seq analysis

Total RNAs were extracted from the *A. missouriensis* wild-type strain as described previously (3). RNA quality and quantity were assessed using a Bioanalyzer DNA1000 (Agilent Technologies, CA, USA). Sequencing libraries were prepared as described previously (22), and sequencing was performed using a NextSeq 500 sequencer (Illumina, CA, USA) to generate directional single-read 75-nucleotide reads. Library construction and sequencing were performed using vertis Biotechnologie AG (Freising-Weihenstephan, Germany). The reads were filtered by sequence quality and mapped to the *A. missouriensis* genome sequence using CLC Genomics Workbench (Illumina).

## RESULTS AND DISCUSSION

### Acyltransferase genes in *A*. *missouriensis*

In the *A. missouriensis* genome, 22 genes have been predicted to encode acyltransferases (7). *In silico* analysis using the Conserved Domain Database (CDD; v3.20; https://www.ncbi.nlm.nih.gov/Structure/cdd/wrpsb.cgi) confirmed the presence of conserved acyltransferase domains in the amino acid sequence of each gene product (Table S2). Ten gene products (AMIS_11360, AMIS_14480, AMIS_30940, AMIS_41550, AMIS_61980, AMIS_70880, AMIS_72650, AMIS_72750, AMIS_73450, and AMIS_75890) harbor the LPLAT_AGPAT-like domain (accession number cd07989 and seven (AMIS_26070, AMIS_50540, AMIS_53620, AMIS_62010, AMIS_62550, AMIS_70460, and AMIS_77300) contain the OafA domain (COG1835. Three gene products (AMIS_11860, AMIS_38540, and AMIS_52390) possess the acyltransferase_3 (AT3) domain (pfam01757). The remaining two gene products, AMIS_760 and AMIS_62890, harbor the branched-chain α-keto acid dehydrogenase subunit E2 (COG0508) and lipid A biosynthesis lauroyl acyltransferase (PRK07920) domains, respectively (Table S2).

Two transcriptional regulators (TcrA and BldC), three sigma factors of the FliA family (FliA1, FliA2, and FliA3), and a sensor histidine kinase (HhkA) work together to regulate the transcription of the developmental genes required for sporangium formation, spore dormancy, and sporangium dehiscence in *A. missouriensis* (17, 20, 23). Therefore, we examined whether the putative acyltransferase genes were under the control of these regulatory proteins. Unexpectedly, according to the RNA-sequencing (RNA-Seq) data obtained in our previous analyses (17, 20, 23), no significant changes in the transcript levels of the 22 genes were detected in the Δ*tcrA*, Δ*hhkA*, Δ*fliA1*Δ*fliA2*Δ*fliA3*, and Δ*bldC* strains compared with the wild-type strain (Table S2), suggesting that these putative acyltransferase genes are not under the control of the regulatory network.

### Effects of the disruption of each putative acyltransferase gene on growth and sporangium formation

**Fig 1:**
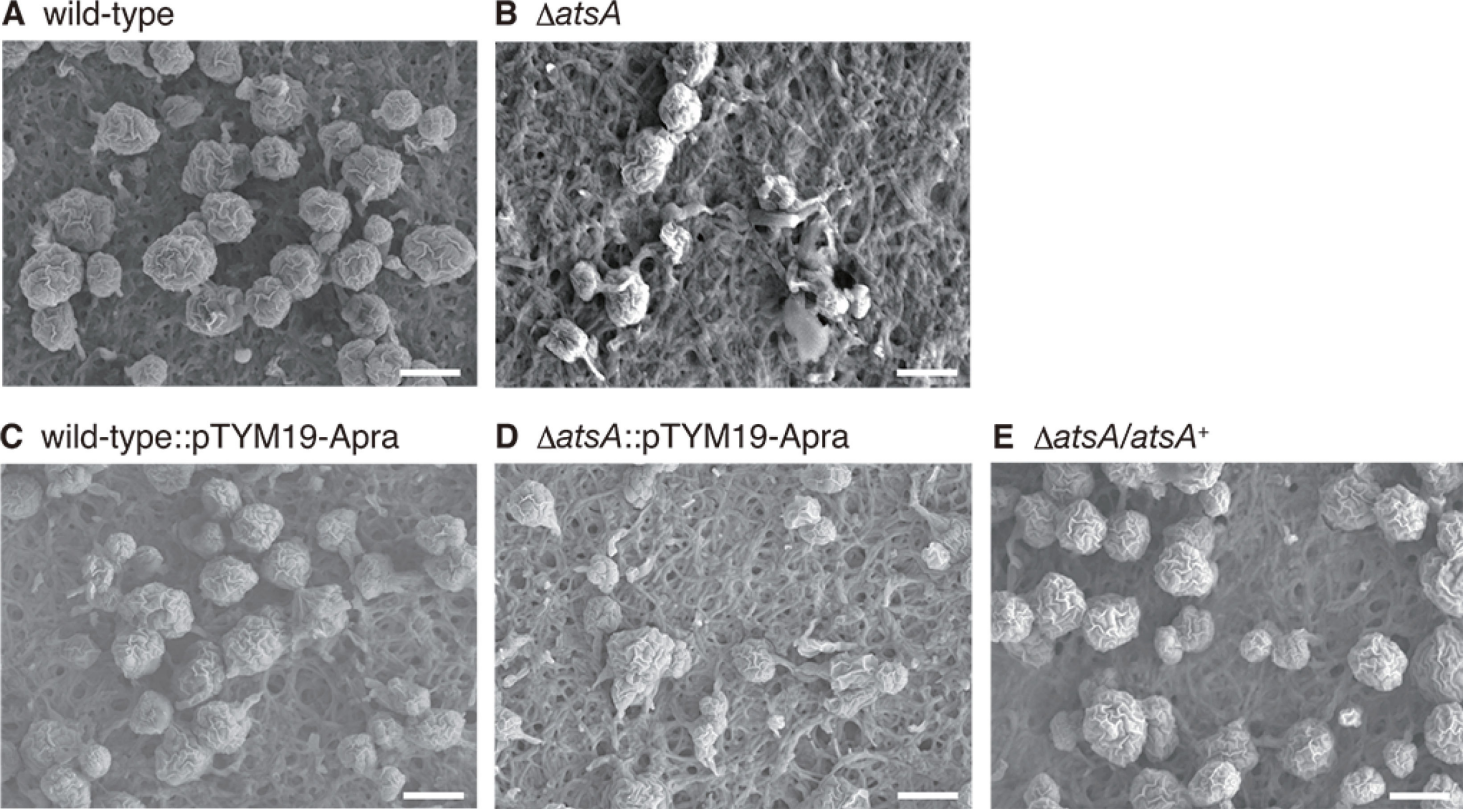
SEM observation of mycelia and sporangia formed on HAT agar after 7 days of cultivation. (**A**) Wild-type strain. (**B**) Δ*atsA* strain. (**C**) Wild-type strain harboring the empty vector pTYM19-Apra. (**D**) Δ*atsA* strain harboring pTYM19-Apra. (**E**) Δ*atsA* strain harboring the complementation plasmid on the chromosome. Scale bars, 5 µm.

**Fig 2:**
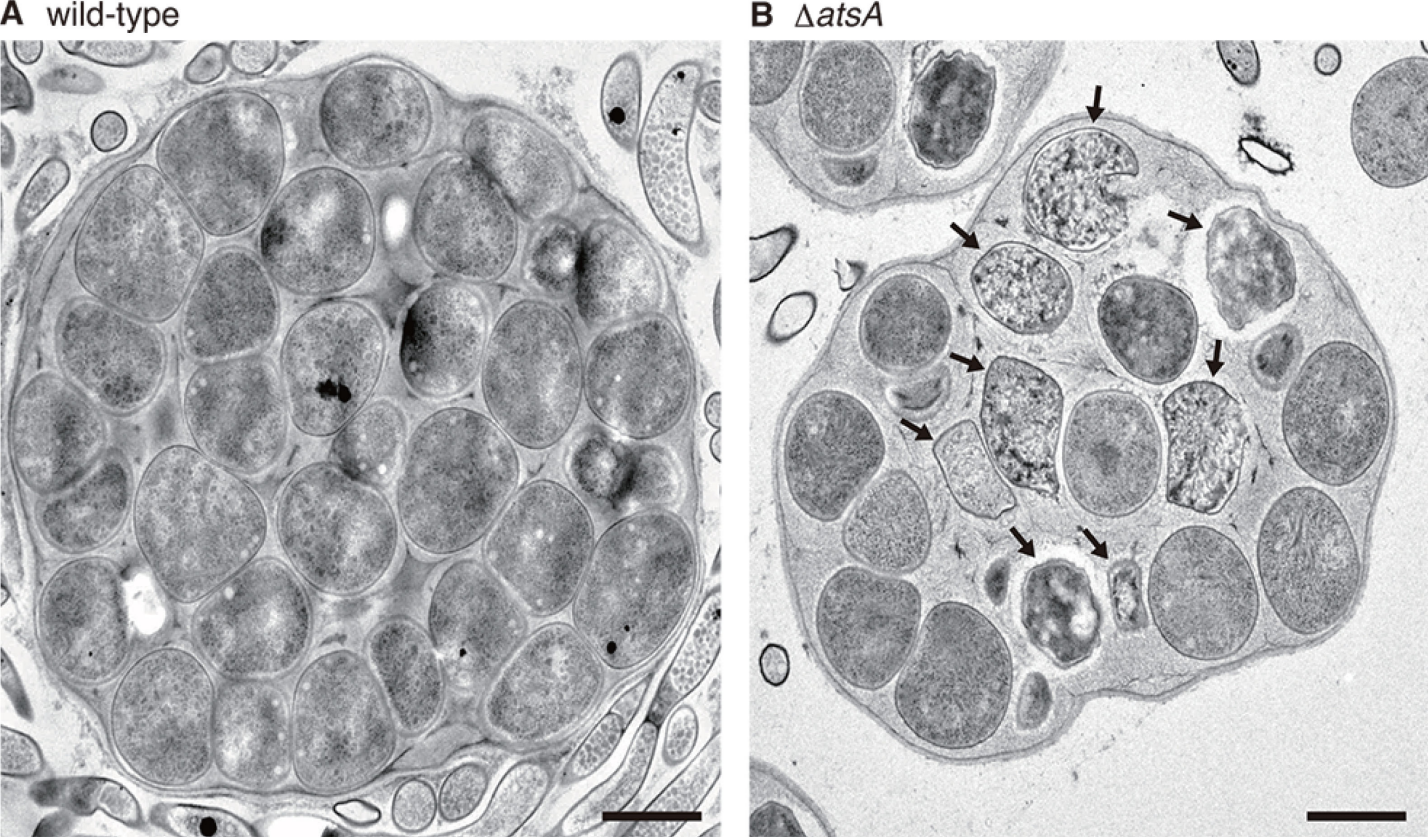
TEM observation of ultrathin sections of the wild-type (**A**) and Δ*atsA* (**B**) sporangia formed on HAT agar after 7 days of cultivation. Abnormal spores in the Δ*atsA* strain are indicated by arrows. Bars, 1 µm.

To investigate the relationship between the putative acyltransferase genes and morphological development in *A. missouriensis*, we inactivated each of the genes by in-frame deletion of coding sequences and compared the phenotypes of the generated mutants with the phenotype of the wild-type strain. Macroscopic observation of mycelia grown on YBNM and HAT agar revealed a growth defect in the *AMIS_11360* null mutant (Δ*AMIS_11360*) strain (data not shown), suggesting that the gene product is required for normal growth under the analyzed culture conditions. Therefore, we excluded the Δ*AMIS_11360* strain from the following analyses in this study (the detailed analysis of *AMIS_11360* will be published elsewhere). However, no difference was observed between the wild-type and other mutant strains in the macroscopic observation of mycelia or sporangia grown on YBNM and HAT agar (data not shown). To further examine sporangium formation in detail, we observed the mycelia and sporangia of the wild-type and mutant strains grown on HAT agar at 30°C for 7 days using SEM. In this experiment, a severe defect in sporangium formation was observed in the *AMIS_52390* null mutant (Δ*AMIS_52390*). In contrast to the wild-type strain, which produced globose or subglobose sporangia, most sporangia in the Δ*AMIS_52390* strain were small or irregular in shape, indicating that the gene product is required for normal sporangium formation (Fig. 1A and B). Hereafter, we refer to AMIS_52390 (450 amino acids) as AtsA (acyltransferase required for sporangium formation) for its putative function. In a gene complementation test, sporangium formation in the Δ*atsA* strain was restored by the introduction of *atsA* with its own promoter on the integration vector pTYM19-Apra (Fig. 1E). In contrast, no phenotypic change was observed when the empty vector was introduced into the wild-type and Δ*atsA* strains (Fig. 1C and D). Meanwhile, no difference was observed between the wild-type and other mutant strains using SEM (Fig. S1). To investigate spore maturation inside the sporangium, TEM was used to observe ultrathin sections of sporangia of the wild-type and Δ*atsA* strains, both of which were grown under the same conditions used for SEM analysis (Fig. 1). Consequently, the wild-type strain generated typical spherical sporangiospores of similar sizes, whereas abnormal cells with irregular shapes were produced in the Δ*atsA* strain, in addition to apparently normal sporangiospores (Fig. 2).

**Fig 3:**
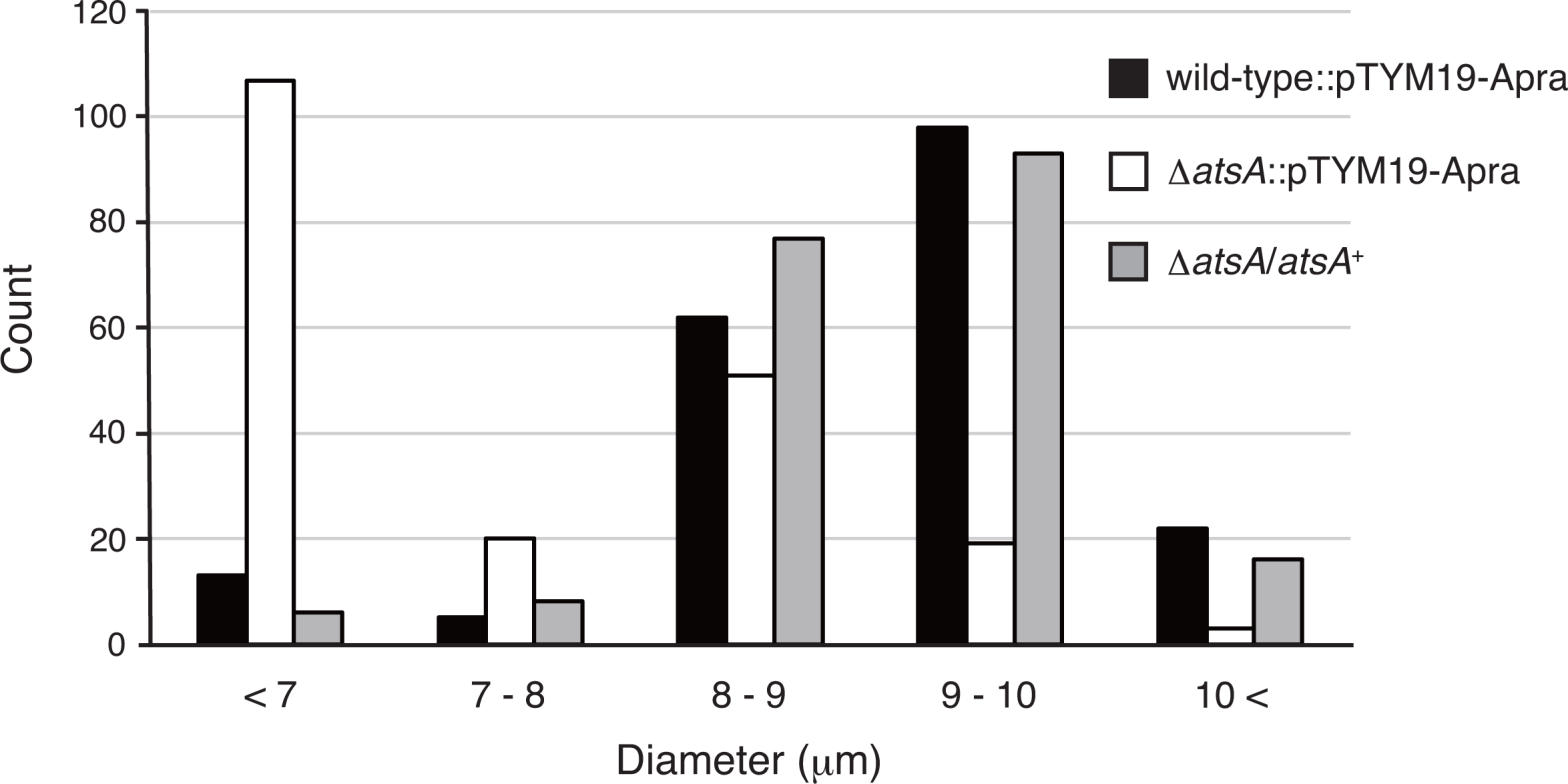
Distribution of the sporangium diameter in the wild-type and Δ*atsA* strains, both of which contained pTYM19-Apra, and the Δ*atsA* strain harboring the complementation plasmid. Each strain was cultivated on HAT agar at 30°C for 7 days for sporangium formation. Sporangia and mycelia harvested from the agar surface were suspended in 50 mM NaCl solution and observed using phase-contrast microscopy. The diameters of 200 sporangia of each strain were measured using micrographs.

To quantitatively evaluate sporangium formation (more specifically, expansion of sporangium), we measured the diameter of sporangia in the wild-type and Δ*atsA* mutant strains, both of which contained pTYM19-Apra, using phase-contrast microscopy. In this experiment, we harvested sporangia and mycelia from sporangium-forming HAT agar and suspended them in 50 mM NaCl solution, in which sporangium dehiscence was not induced (our unpublished result). Whereas the sporangia in the wild-type strain typically exhibited a diameter ranging from 8 to 10 µm, more than half of the sporangia in the Δ*atsA* strain displayed a reduced diameter of less than 7 µm (Fig. 3). Meanwhile, the diameter of approximately one-third of the sporangia in the Δ*atsA* strain ranged between 8 and 10 µm (Fig. 3). This result clearly indicated a significant increase in the number of smaller sporangia in the Δ*atsA* strain compared with the wild-type strain. The *atsA* complementation strain produced normal sporangia, similar to the wild-type strain (Fig. 3). These results demonstrate that *atsA* is required for normal sporangium formation under the culture conditions tested.

### Effects of the disruption of each putative acyltransferase gene on sporangium dehiscence

**Fig 4:**
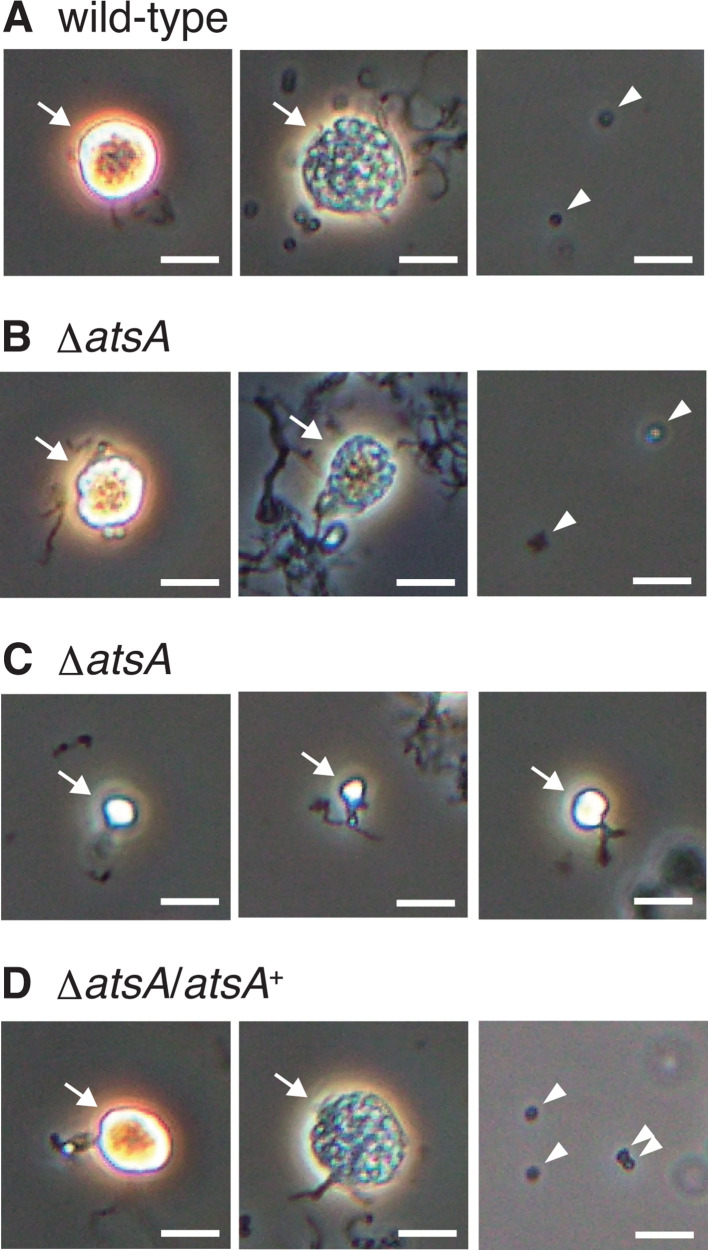
Observation of sporangium dehiscence using phase-contrast microscopy. Sporangia formed on HAT agar were harvested and suspended in 25 mM histidine solution to induce sporangium dehiscence. Micrographs of the wild-type strain (**A**), the Δ*atsA* strain (**B and C**), and the Δ*atsA* strain harboring the complementation plasmid (**D**) are shown. The left panels of each strain show the images taken immediately after the suspension. The middle and right panels show images taken 30 and 60 min after the suspension, respectively. The panels in (**B**) and (**C**) show normal and immature (small) sporangia of the Δ*atsA* strain, respectively. Sporangia (including transparent and immature sporangia) and zoospores are indicated by arrows and arrowheads, respectively. Bars, 5 µm. The entire images of each microscopic field are shown in Fig. S2.

We examined sporangium dehiscence and the motility of zoospores released from sporangia using phase-contrast microscopy. After cultivation on HAT agar at 30°C for 7 days, sporangia formed on the agar were harvested and suspended in 25 mM histidine solution. The suspensions were incubated with rotation at room temperature for 1 h to induce sporangium dehiscence. Under these conditions, the wild-type sporangia appeared phase-bright immediately after suspension, and the sporangium membrane gradually became transparent before spore release (Fig. 4A; Fig. S2A). In the Δ*atsA* strain, sporangia with normal shapes opened and released spores similarly to those of the wild-type strain, whereas the dehiscence process did not proceed, and the spores were not released in the small sporangia (Fig. 4B and C; Fig. S2B). Sporangia of the *atsA* complementation strain opened and released spores similarly to the wild-type sporangia (Fig. 4D; Fig. S2C). Meanwhile, sporangia of the other mutant strains opened normally and released motile zoospores (Fig. S3), showing that each of the remaining acyltransferase genes is not essential for sporangium dehiscence and zoospore motility under the conditions analyzed in this experiment.

**Fig 5:**
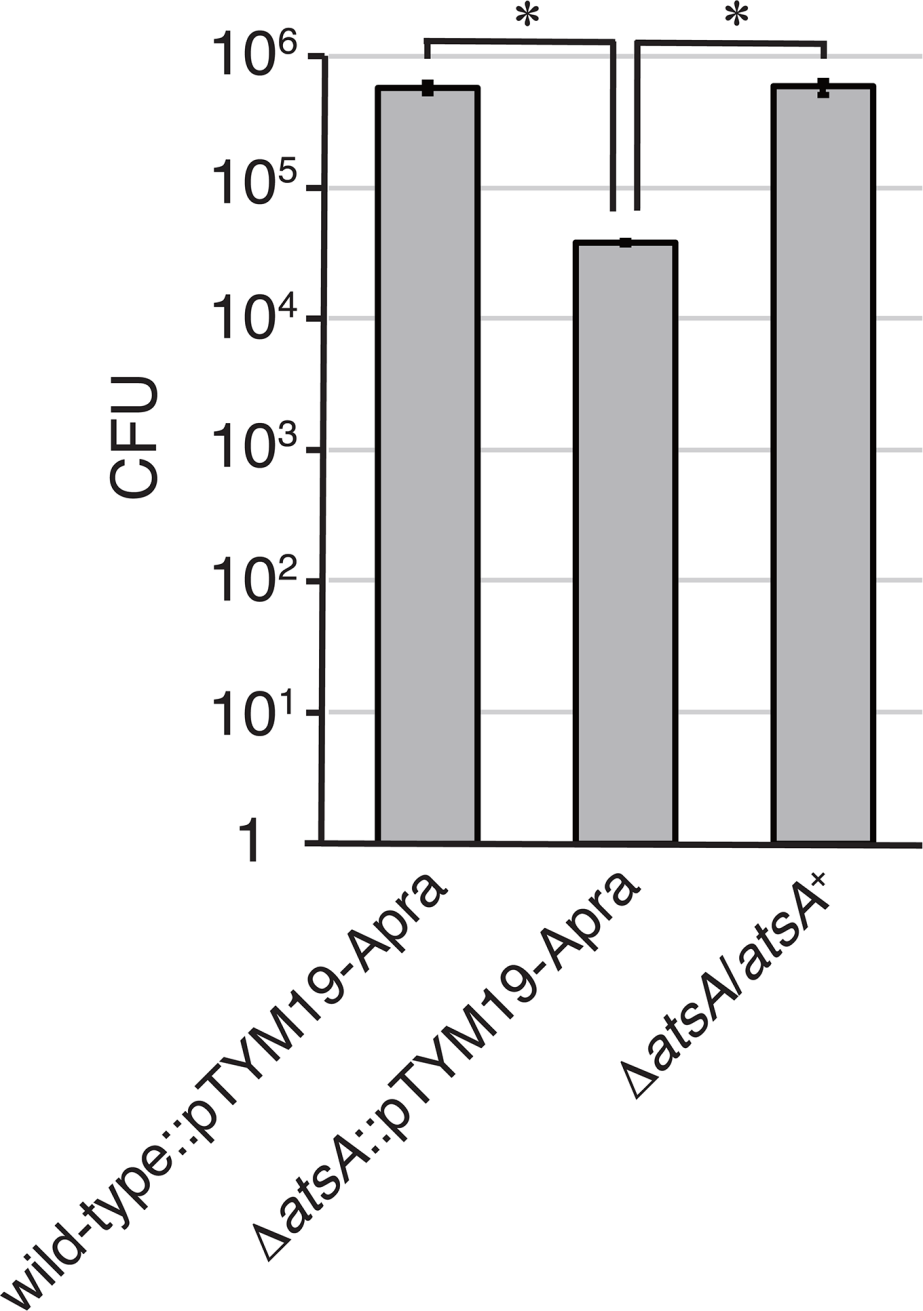
The number of zoospores released from sporangia of the wild-type and Δ*atsA* strains, both of which contained pTYM19-Apra, and the Δ*atsA* strain harboring the complementation plasmid. Each strain was cultivated on HAT agar at 30°C for 7 days to form sporangia, and 25 mM NH_4_HCO_3_ solution was poured onto the agar plate to induce sporangium dehiscence. The zoospore suspension was collected from the agar plate and filtered through a membrane filter to eliminate hyphae and sporangia. A portion of the filtrate was inoculated onto a YBNM agar plate, and the plate was incubated at 30°C for 2 days to form colonies. The number of zoospores released from sporangia was estimated from the colony-forming units (CFUs) on YBNM agar. Zoospores were collected 60 min after pouring the NH_4_HCO_3_ solution. The values represent the mean ± SE of three biological replicates. Statistically significant differences (*P* value < 0.05) between the two strains are marked with asterisks.

Next, we quantified spores released from the sporangia of the wild-type and mutant strains. In this experiment, 10 mL of 25 mM NH_4_HCO_3_ solution was poured onto a sporangium-forming HAT agar plate, and the plate was incubated at room temperature for 1 h to release zoospores from sporangia. Then, the zoospore suspension was collected from the plate and filtered through a 5 µm membrane filter to eliminate hyphae and sporangia. A portion of the filtrate was inoculated on YBNM agar plates, and the plates were incubated at 30°C for 2 days to form colonies. Consistent with the observations by SEM and phase-contrast microscopy, the number of colonies was almost identical between the wild-type and mutant strains, except for the Δ*atsA* strain, which also indicates that none of the genes other than *atsA* is essential for sporangium formation and dehiscence (Fig. S4). Meanwhile, the Δ*atsA* strain formed fewer colonies than the wild-type strain (Fig. 5). Although the wild-type strain produced over 10^5^ colonies per HAT agar plate, the Δ*atsA* strain formed over 10^4^ colonies under the same conditions (Fig. 5). In this experiment, the wild-type and Δ*atsA* strains harbored pTYM19-Apra on their chromosomes. This phenotypic change was also restored in the *atsA* complementation strain (Fig. 5). We assumed two possible reasons for the reduced number of colonies of the Δ*atsA* strain: (i) the small sporangia of the Δ*atsA* strain cannot open to release spores (Fig. 4C) and (ii) abnormal spores inside Δ*atsA* sporangia (Fig. 2B) do not seem to germinate to form colonies on YBNM agar.

### *In silico* and transcriptional analyses of *atsA*

**Fig 6:**
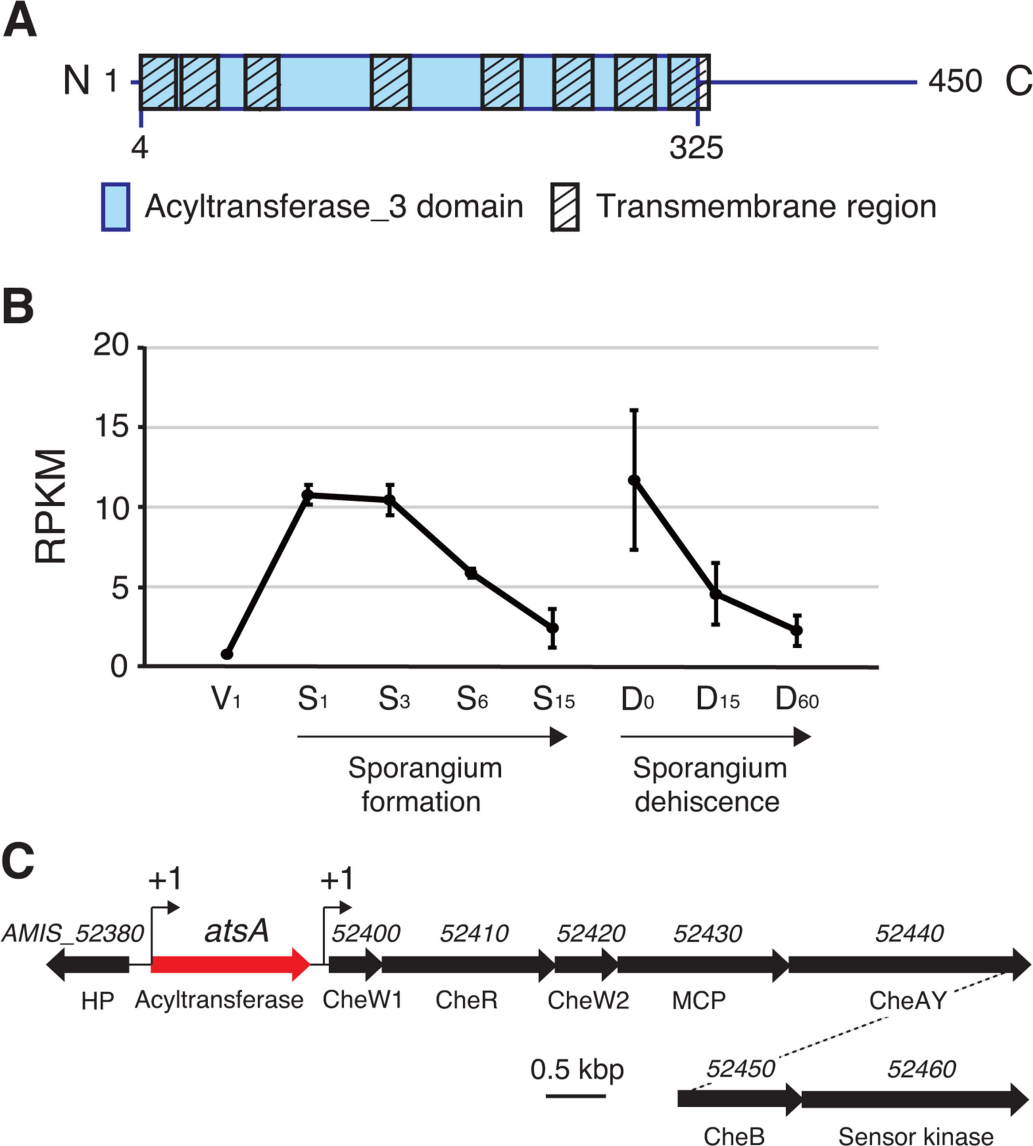
Predicted domain structure of AtsA and transcriptional profile and gene organization of *atsA*. (**A**) Schematic representation of the domain structure of AtsA. (**B**) Transcriptional profile of *atsA*. Data are the average number of reads per kilobase of coding sequence per million mapped reads (RPKMs) in the wild-type strain by RNA-seq analysis under various culture conditions. RNA samples were prepared from substrate hyphae grown on YBNM agar for 24 h (**V_1_**) or HAT agar for 24 h (**S_1_**), mixtures of substrate hyphae and sporangia grown on HAT agar for 3, 6, and 15 days (S_3_, S_6_, and S_15_, respectively), and sporangia (including some substrate hyphae) incubated in 25 mM histidine solution for sporangium dehiscence for 0, 15, and 60 min (D_0_, D_15_, and D_60_, respectively). Average RPKM values ± SEs for the three biological replicates are shown. (**C**) Gene organization of *atsA*. The arrows indicate the locations of the open reading frames, including their length and direction. Gene identifiers and putative gene products are shown above and below the arrows, respectively. HP, hypothetical protein; MCP, methyl-accepting chemotaxis protein; CheAY, fusion protein of CheA and CheY.

As described above, the protein database search proposed that AtsA harbors an AT3 domain (residues 4–325), which is present in a wide range of acyltransferases, including bacterial enzymes such as OatA in *Listeria monocytogenes* and *Staphylococcus aureus*, and NolL in *Mesorhizobium loti* (24–27). AT3 proteins are categorized into a family of integral membrane proteins that catalyze acylation (more specifically, acetylation in most cases) of cell surface structures including lipopolysaccharides, peptidoglycans, and capsules (28). Most frequently, the AT3 domain contains 10 transmembrane helices (28). The SOSUI engine (ver. 1.11; https://harrier.nagahama-i-bio.ac.jp/sosui/mobile/) predicted that AtsA is an integral membrane protein with eight transmembrane regions. Thus, AtsA was predicted to be a stand-alone AT3 domain-containing protein with an unusual number of transmembrane helices (Fig. 6A). In the actinomycete *Corynebacterium glutamicum*, the AT3 protein TmaT acetylates trehalose corynomycolates in the biosynthetic process of its complex cell walls, which are rich in long-chain mycolic acids (29). AtsA and TmaT share an amino acid sequence identity of 13% (Fig. S5). Thus, AtsA may be involved in the acylation of fatty acids or sugar moieties of cell surface structures during sporangium formation rather than in the remodeling of membrane lipid composition.

To analyze the time points at which *atsA* transcription occurs, we referred to an exhaustive RNA-seq analysis, which was performed previously under various culture conditions covering the life cycle of *A. missouriensis* (30). For this analysis, RNAs were prepared from the substrate hyphae cultivated on YBNM agar at 30°C for 24 h and from the mycelia and/or sporangia grown on HAT agar for 1, 3, 6, and 15 days for sporangium formation. For sporangium dehiscence, RNAs were prepared from sporangia (including some substrate hyphae) suspended and incubated in 25 mM histidine solution for 0, 15, and 60 min. RNA samples were prepared in triplicate at each time point. Almost no *atsA* transcripts were detected in the substrate hyphae cultivated on YBNM agar at 30°C for 24 h (Fig. 6B; V_1_). While weak transcripts were detected in the substrate hyphae and/or sporangia cultivated on HAT agar at 30°C for 1 and 3 days, it gradually decreased during sporangium formation (Fig. 6B; S_1_, S_3_, S_6_, and S_15_). A similar decrease in the transcript levels was observed during sporangium dehiscence (Fig. 6B; D_0_, D_15_, and D_60_). We also compared the transcript levels (reads per kilobase of coding sequence per million mapped reads values) of *atsA* with those of the other putative acyltransferase genes analyzed by gene disruption (Fig. S6). Based on the transcriptional profiles, *atsA* was regarded as a weakly expressed gene because its transcript levels were very low among the 22 genes (Fig. S6). However, it is difficult to determine the relationship between AtsA and sporangium formation based on the expression pattern of *atsA*.

We also performed a Cappable-seq analysis to comprehensively determine the transcriptional start points using the RNA samples extracted at the same time points used for the exhaustive RNA-seq analysis (22). According to this analysis, the transcriptional start site of *atsA* was determined to be one nucleotide upstream of the translational start codon, showing that *atsA* is transcribed as a leaderless messenger RNA (Fig. 6C). In addition, *atsA* is presumably transcribed as a monocistronic transcript because a transcriptional start site was detected within the intergenic region between *atsA* and the downstream *AMIS_52400* gene, the first gene of one of the three chemotaxis gene clusters of this species, named *che* 3 (Fig. 6C), which did not seem to be related to zoospore chemotaxis (our unpublished result).

### Conclusion

In the present study, we demonstrated that *atsA* is required for normal sporangium formation in *A. missouriensis*. We assume that *atsA* is involved in spore maturation within a sporangium because abnormal sporangiospores were observed in the Δ*atsA* strain (Fig. 2B). We anticipate that the involvement of AtsA in sporangium formation is widespread in members of the genus *Actinoplanes* because the orthologs of AtsA are highly conserved among 46 *Actinoplanes* bacteria whose genome sequences and gene annotations have been registered in the NCBI genome database (https://www.ncbi.nlm.nih.gov/genome/; Fig. S7). The molecular mechanism of sporangium formation in *A. missouriensis* remains to be elucidated; however, exploration of the enzymatic activity and physiological role of AtsA, a putative acyltransferase, will provide key clues. It should be noted that there remains a possibility that a gene(s) other than the 22 genes analyzed in this study also encodes a functional acyltransferase that possesses a domain with low similarity to the conserved acyltransferase domains or a novel acyltransferase domain in *A. missouriensis*. Considering the possibility that acyltransferases play redundant roles in morphological development, the construction and phenotypic analysis of null mutant strains of multiple acyltransferase genes are significant subjects for future research.

## Data Availability

Nucleotide sequence data from the Cappable-Seq analysis have been deposited in the DDBJ Sequence Read Archive under the accession number DRA012687.

## SUPPLEMENTAL MATERIAL

Supplemental material spectrum.04010-23-s0001.pdf Fig. S1-S7; Tables S1 and S2.

ASM does not own the copyrights to Supplemental Material that may be linked to, or accessed through, an article. The authors have granted ASM a non-exclusive, world-wide license to publish the Supplemental Material files. Please contact the corresponding author directly for reuse.
