# Supplementary material for: Involvement of a putative acyltransferase gene in sporangium formation in Actinoplanes missouriensis
Source: Microbiol Spectr. 2024 Mar 19;12(5):e04010-23. doi: 10.1128/spectrum.04010-23 (PMC11064477; doi:10.1128/spectrum.04010-23)
Supplement: Supplemental material — Fig. S1-S7; Tables S1 and S2. [file spectrum.04010-23-s0001.pdf]

Involvement of a putative acyltransferase gene in sporangium formation in *Actinoplanes missouriensis*

Shixuan Hu,[1] Satoshi Maeda,[1] Takeaki Tezuka,[1,2,*] Yasuo Ohnishi[1,3,*]

[1]Department of Biotechnology, Graduate School of Agricultural and Life Sciences, The University of Tokyo, Bunkyo-ku, Tokyo, Japan

[2]Graduate School of Infection Control Sciences, Kitasato University, Minato-ku, Tokyo, Japan

[3]Collaborative Research Institute for Innovative Microbiology, The University of Tokyo, Bunkyo-ku, Tokyo, Japan

*Address correspondence to Takeaki Tezuka, atezuka@mail.ecc.u-tokyo.ac.jp; Yasuo Ohnishi, ayasuo@mail.ecc.u-tokyo.ac.jp

Contents

Fig. S1. SEM observation of mycelia and sporangia in acyltransferase gene mutant strains

Fig. S2. Observation of sporangium dehiscence using phase-contrast microscopy

Fig. S3. Observation of sporangium dehiscence in acyltransferase gene mutants by phase-contrast microscopy

Fig. S4. The number of zoospores released from the sporangia

Fig. S5. Amino acid sequence alignment of AtsA and TmaT

Fig. S6. Transcript levels of the 22 putative acyltransferase genes

Fig. S7. Amino acid sequence alignment of AtsA and its orthologs from 46 *Actinoplanes* bacteria

Table S1. Primers used in this study

Table S2. Putative acyltransferase genes in the *A. missouriensis* genome

Supplemental references

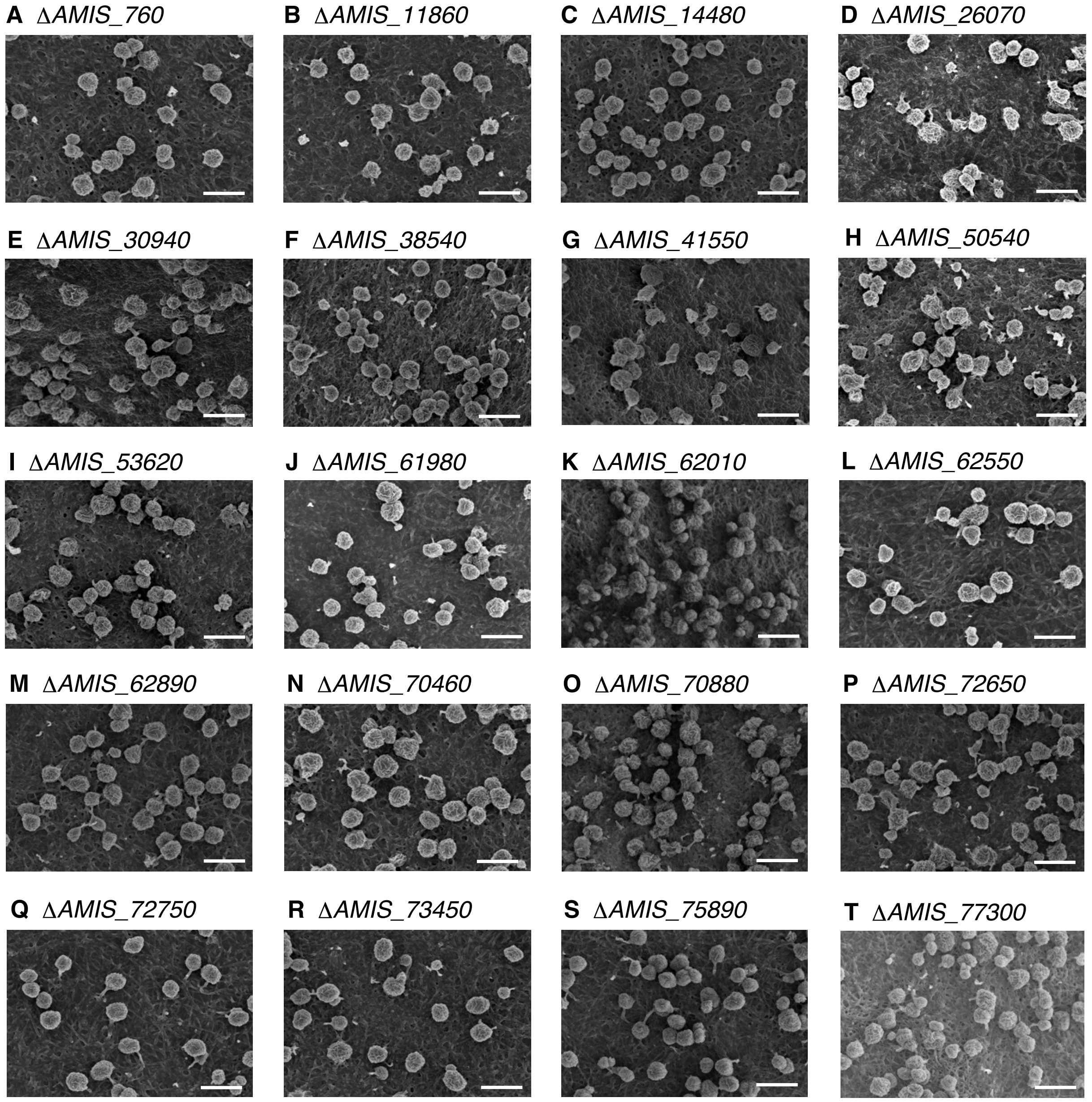

Fig. S1. SEM observation of mycelia and sporangia in acyltransferase gene mutant strains. Each strain was cultivated on HAT agar at 30°C for 7 days. Scale bars, 10 μm. (**A**) Δ*AMIS_760* strain. (**B**) Δ*AMIS_11860* strain. (**C**) Δ*AMIS_14480* strain. (**D**) Δ*AMIS_26070* strain. (**E**) Δ*AMIS_30940* strain. (**F**) Δ*AMIS_38540* strain. (**G**) Δ*AMIS_41550* strain. (**H**) Δ*AMIS_50540* strain. (**I**) Δ*AMIS_53620* strain. (**J**) Δ*AMIS_61980* strain. (**K**) Δ*AMIS_62010* strain. (**L**) Δ*AMIS_62550* strain. (**M**) Δ*AMIS_62890* strain. (**N**) Δ*AMIS_70460* strain. (**O**) Δ*AMIS_70880* strain. (**P**) Δ*AMIS_72650* strain. (**Q**) Δ*AMIS_72750* strain. (**R**) Δ*AMIS_73450* strain. (**S**) Δ*AMIS_75890* strain. (**T**) Δ*AMIS_77300* strain.

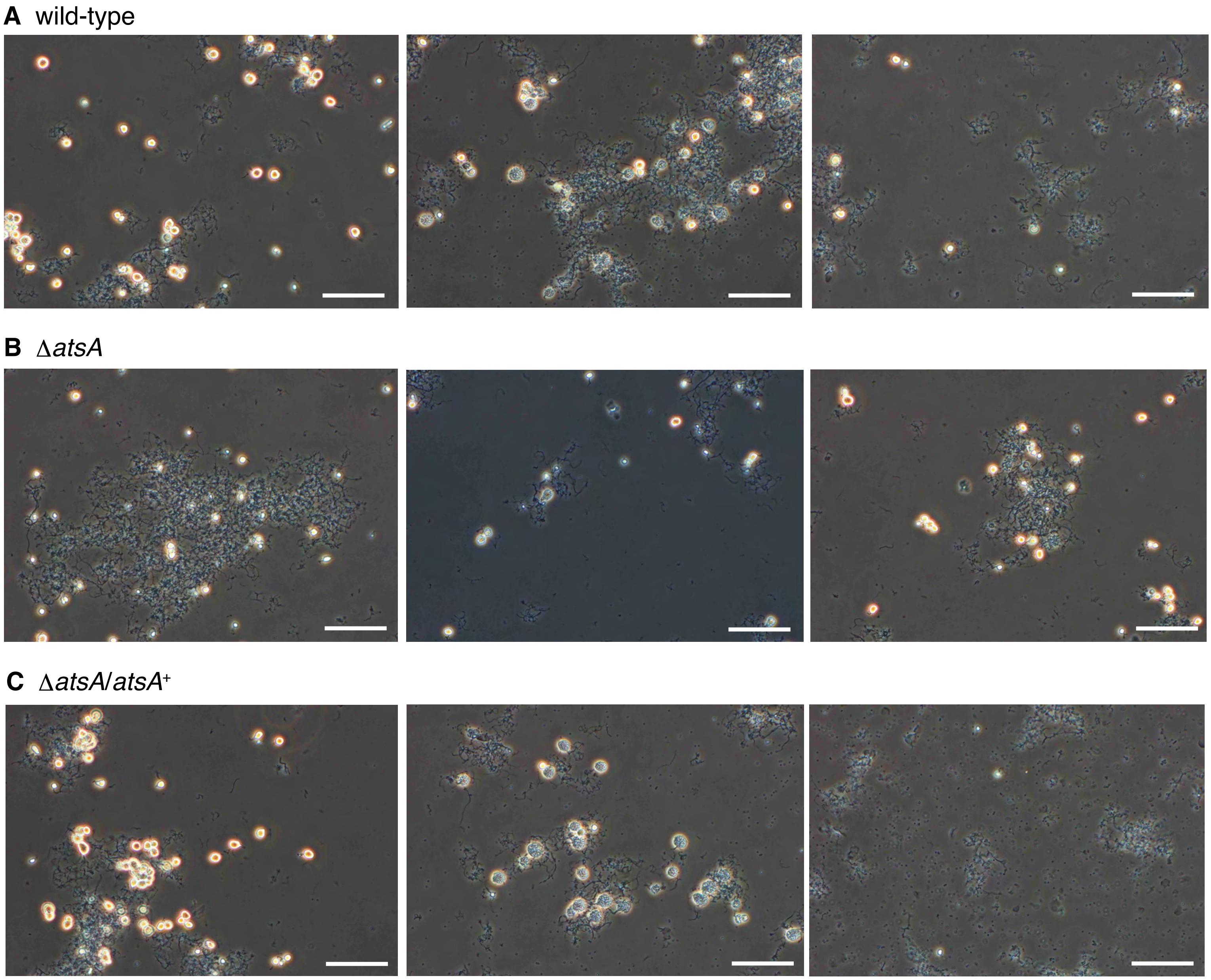

Fig. S2. Observation of sporangium dehiscence using phase-contrast microscopy. Sporangia formed on HAT agar were harvested and suspended into 25 mM histidine solution to induce sporangium dehiscence. Micrographs of the wild-type strain (**A**), the Δ*atsA* strain (**B**), and the Δ*atsA* strain harboring the complementation plasmid (**C**) are shown. Each panel is the entire image of the microscopic field shown in Fig. 4 (the panels in Figs. 4B and 4C were generated from the panels in Fig. S2B). Bars, 50 μm.

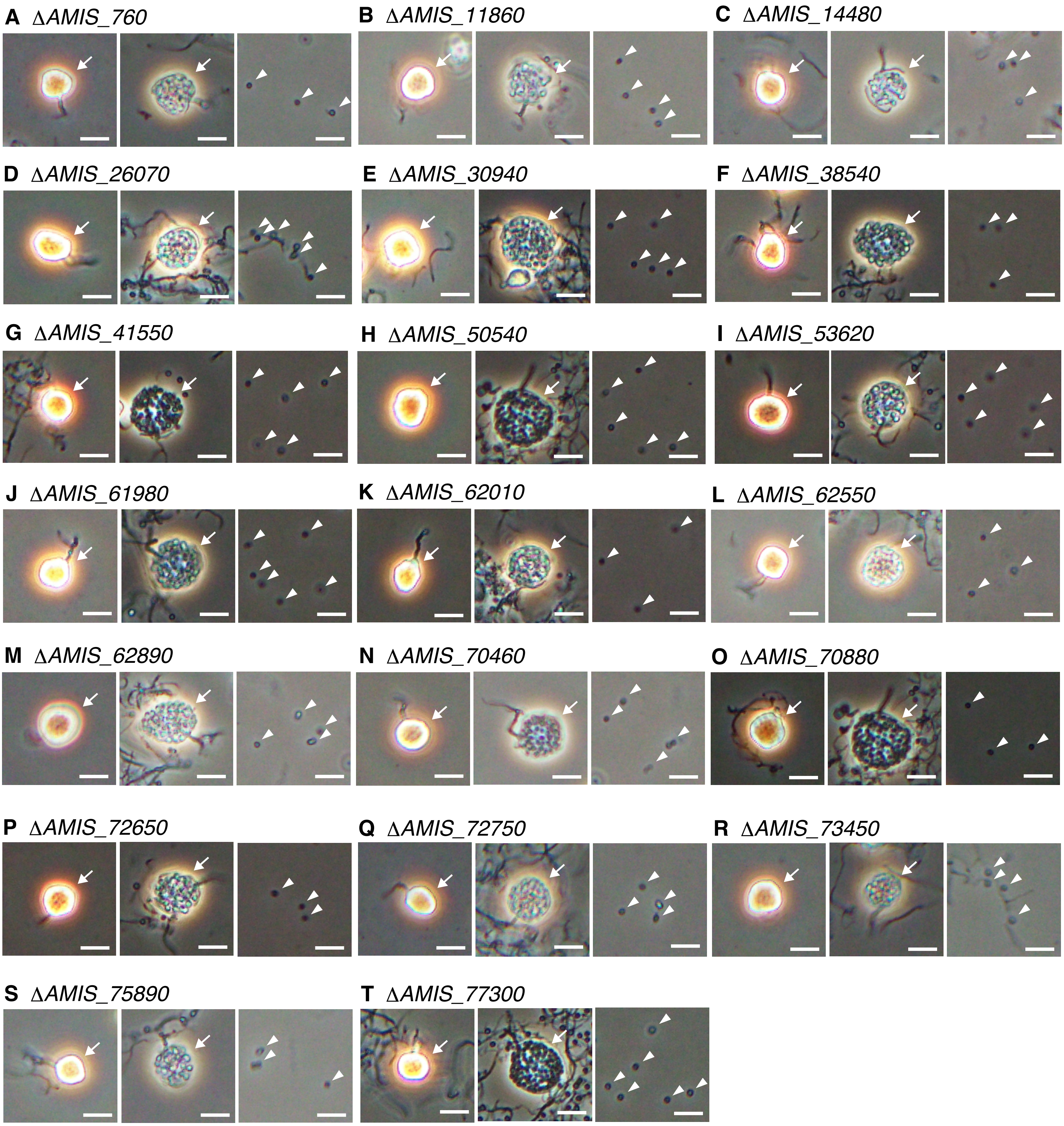

Fig. S3. Observation of sporangium dehiscence in acyltransferase gene mutants by phase-contrast microscopy. Sporangia formed on HAT agar were harvested and suspended in 25 mM histidine solution to induce sporangium dehiscence. Microscopic images of mutant strains are shown. The left panels of each strain show the images taken immediately after the suspension. The middle and right panels show images taken 30 and 60 min after the suspension, respectively. Sporangia and zoospores are indicated by arrows and arrowheads, respectively. Bars, 5 µm. (**A**) Δ*AMIS_760* strain. (**B**) Δ*AMIS_11860* strain. (**C**) Δ*AMIS_14480* strain. (**D**) Δ*AMIS_26070* strain. (**E**) Δ*AMIS_30940* strain. (**F**) Δ*AMIS_38540* strain. (**G**) Δ*AMIS_41550* strain. (**H**) Δ*AMIS_50540* strain. (**I**) Δ*AMIS_53620* strain. (**J**) Δ*AMIS_61980* strain. (**K**) Δ*AMIS_62010* strain. (**L**) Δ*AMIS_62550* strain. (**M**) Δ*AMIS_62890* strain. (**N**) Δ*AMIS_70460* strain. (**O**) Δ*AMIS_70880* strain. (**P**) Δ*AMIS_72650* strain. (**Q**) Δ*AMIS_72750* strain. (**R**) Δ*AMIS_73450* strain. (**S**) Δ*AMIS_75890* strain. (**T**) Δ*AMIS_77300* strain.

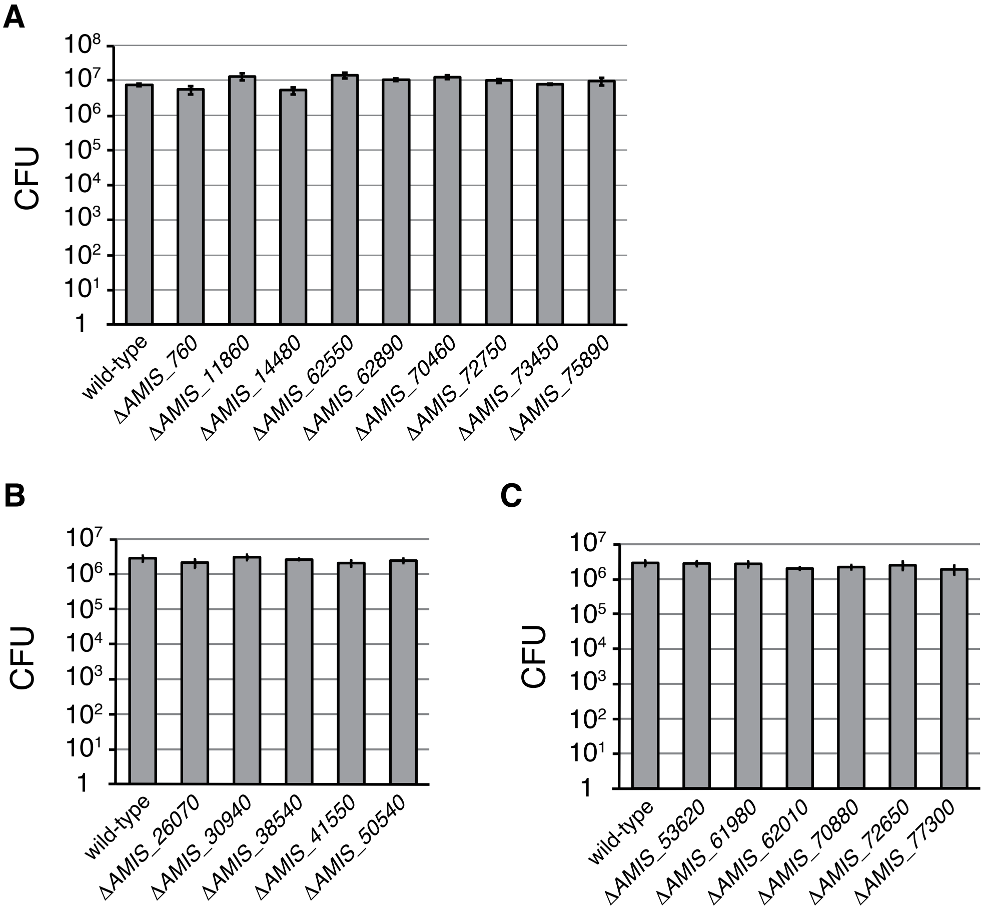

Fig. S4. The number of zoospores released from the sporangia. Each strain was cultivated on HAT agar at 30°C for 7 days. Zoospores released from sporangia were counted as colony-forming units (CFUs) formed on YBNM agar. The values represent the mean ± standard error of three biological replicates. (**A**) The wild-type and nine mutant (Δ*AMIS_760*, Δ*AMIS_11860*, Δ*AMIS_14480*, Δ*AMIS_62550*, Δ*AMIS_62890*, Δ*AMIS_70460*, Δ*AMIS_72750*, Δ*AMIS_73450*, and Δ*AMIS_75890*) strains. (**B**) The wild-type and five mutant (Δ*AMIS_26070*, Δ*AMIS_30940*, Δ*AMIS_38540*, Δ*AMIS_41550*, and Δ*AMIS_50540*) strains. (**C**) The wild-type and six mutant (Δ*AMIS_53620*, Δ*AMIS_61980*, Δ*AMIS_62010*, Δ*AMIS_70880*, Δ*AMIS_72650*, and Δ*AMIS_77300*) strains.

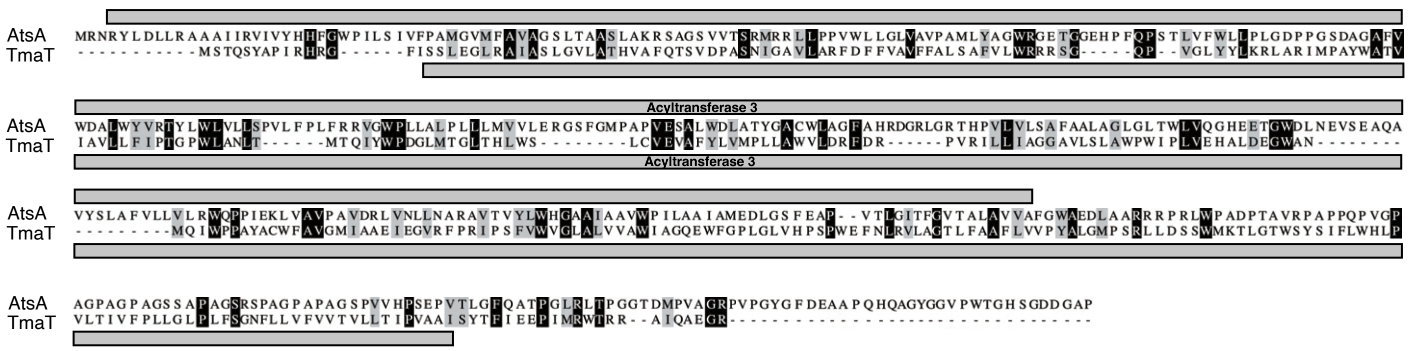

Fig. S5. Amino acid sequence alignment of AtsA and TmaT. The sequences share 13% identity. The acyltransferase_3 domains predicted in AtsA and TmaT are shown by gray rectangles above and below the alignment, respectively.

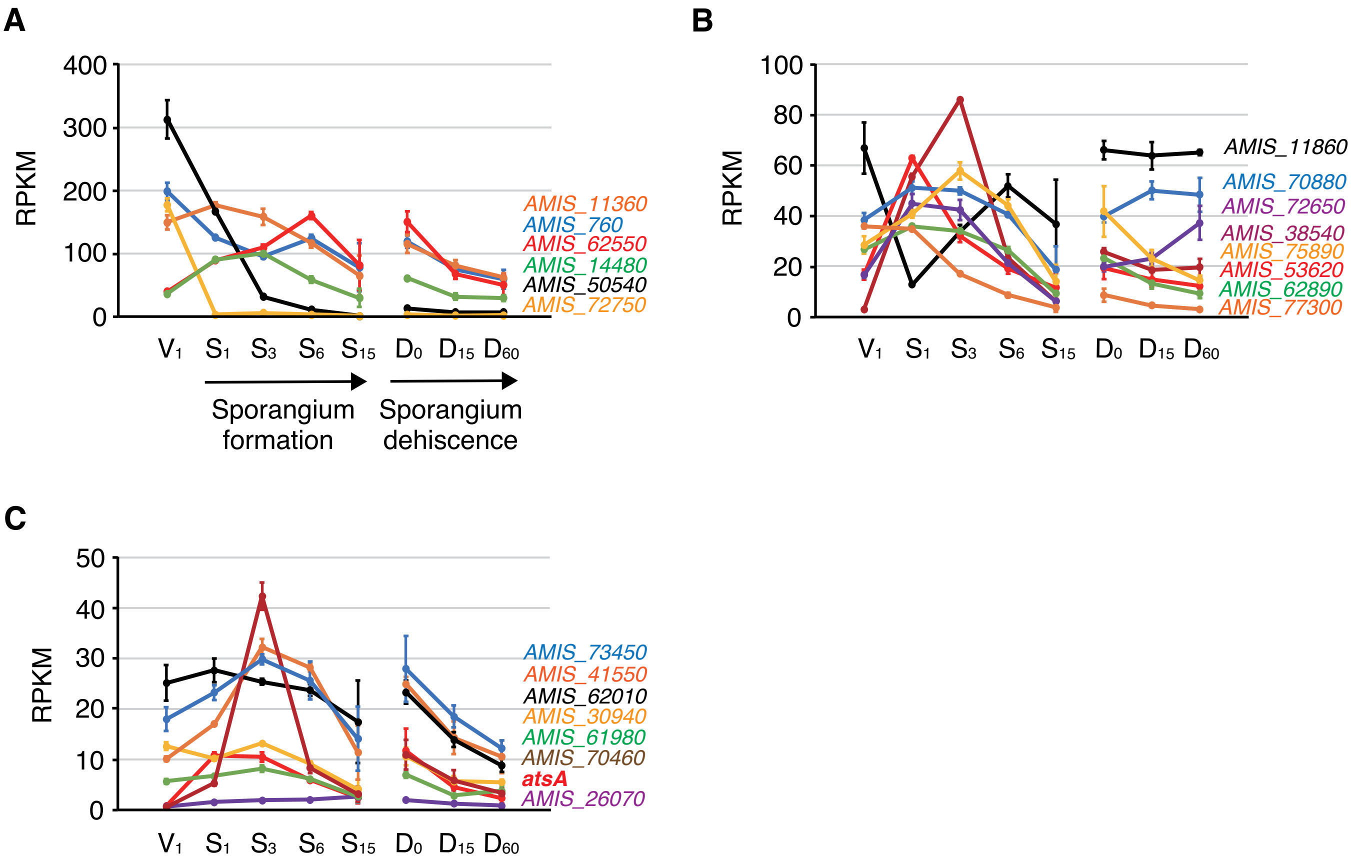

Fig. S6. Transcript levels of the 22 putative acyltransferase genes. (**A**) Transcriptional profiles of six genes (*AMIS_760*, *AMIS_11360*, *AMIS_14480*, *AMIS_50540*, *AMIS_62550*, and *AMIS_72750*) with relatively high transcript levels. (**B**) Transcriptional profiles of eight genes (*AMIS_11860*, *AMIS_38540*, *AMIS_53620*, *AMIS_62890*, *AMIS_70880*, *AMIS_72650*, *AMIS_75890*, and *AMIS_77300*) with relatively low transcript levels. (**C**) Transcriptional profiles of eight genes (*atsA*, *AMIS_26070*, *AMIS_30940*, *AMIS_41550*, *AMIS_61980*, *AMIS_62010*, *AMIS_70460*, and *AMIS_73450*) with very low transcript levels. RNA samples were prepared as described in Fig. 6B. Average RPKM values ± standard errors for the three biological replicates are shown.

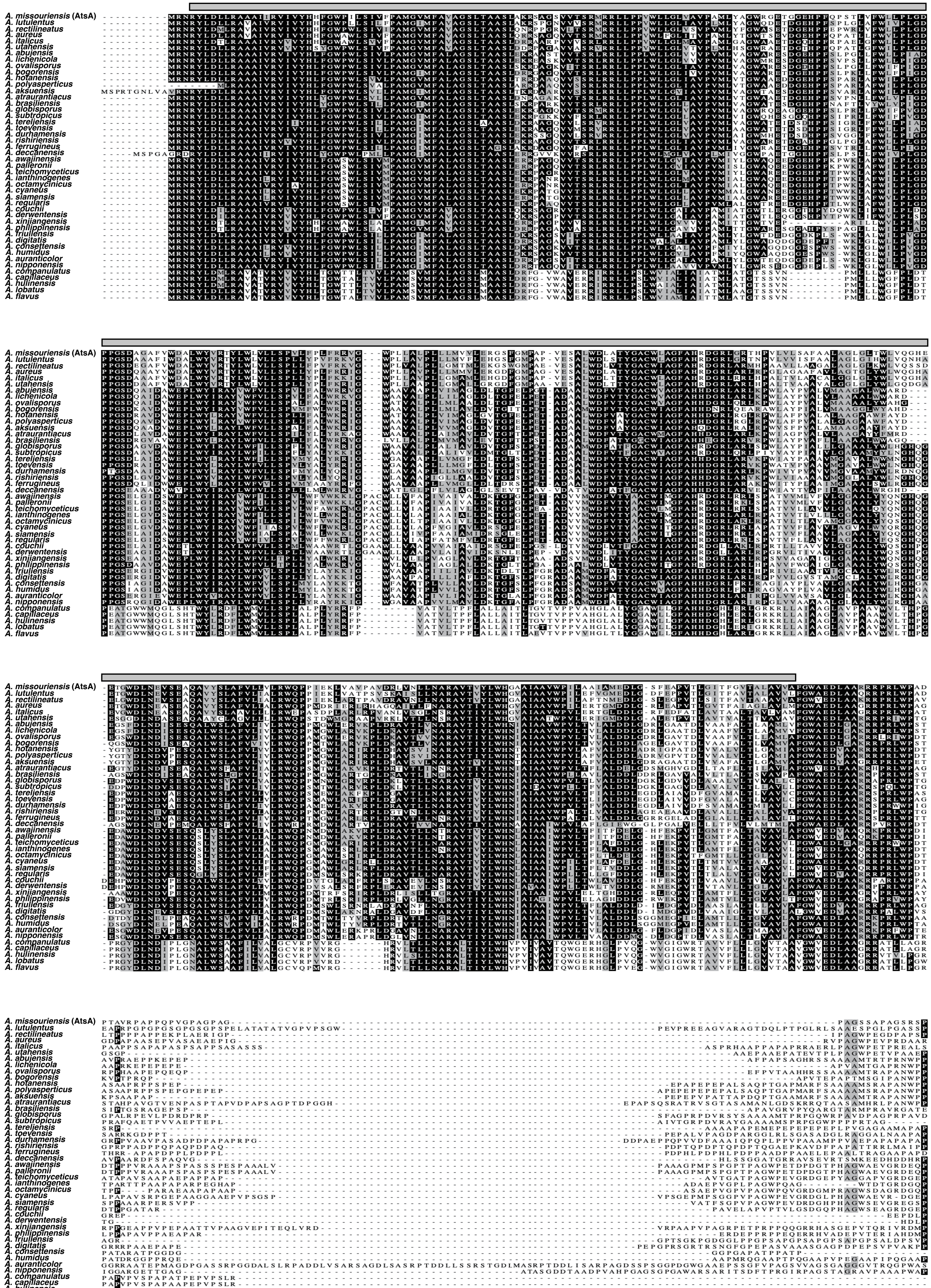
A. missouriensis (AtsA)
A. lutulentus
A. rectilineatus
A. aureus
A. italicus
A. utahensis
A. abujensis
A. lichenicola
A. ovalisporus
A. bogorensis
A. hotanensis
A. polyasperticus
A. aksuensis
A. atraurantiacus
A. brasiliensis
A. globisporus
A. subtropicus
A. tereljensis
A. toevensis
A. durhamensis
A. rishiriensis
A. ferrugineus
A. deccanensis
A. awajinensis
A. palleronii
A. teichomyceticus
A. ianthinogenes
A. octamycinicus
A. cyaneus
A. siamensis
A. regularis
A. couchii
A. derwentensis
A. xinjiangensis
A. philippinensis
A. friuliensis
A. digitatis
A. consettensis
A. humidus
A. auranticolor
A. nipponensis
A. companulatus
A. capillaceus
A. hulinensis
A. lobatus
A. flavus

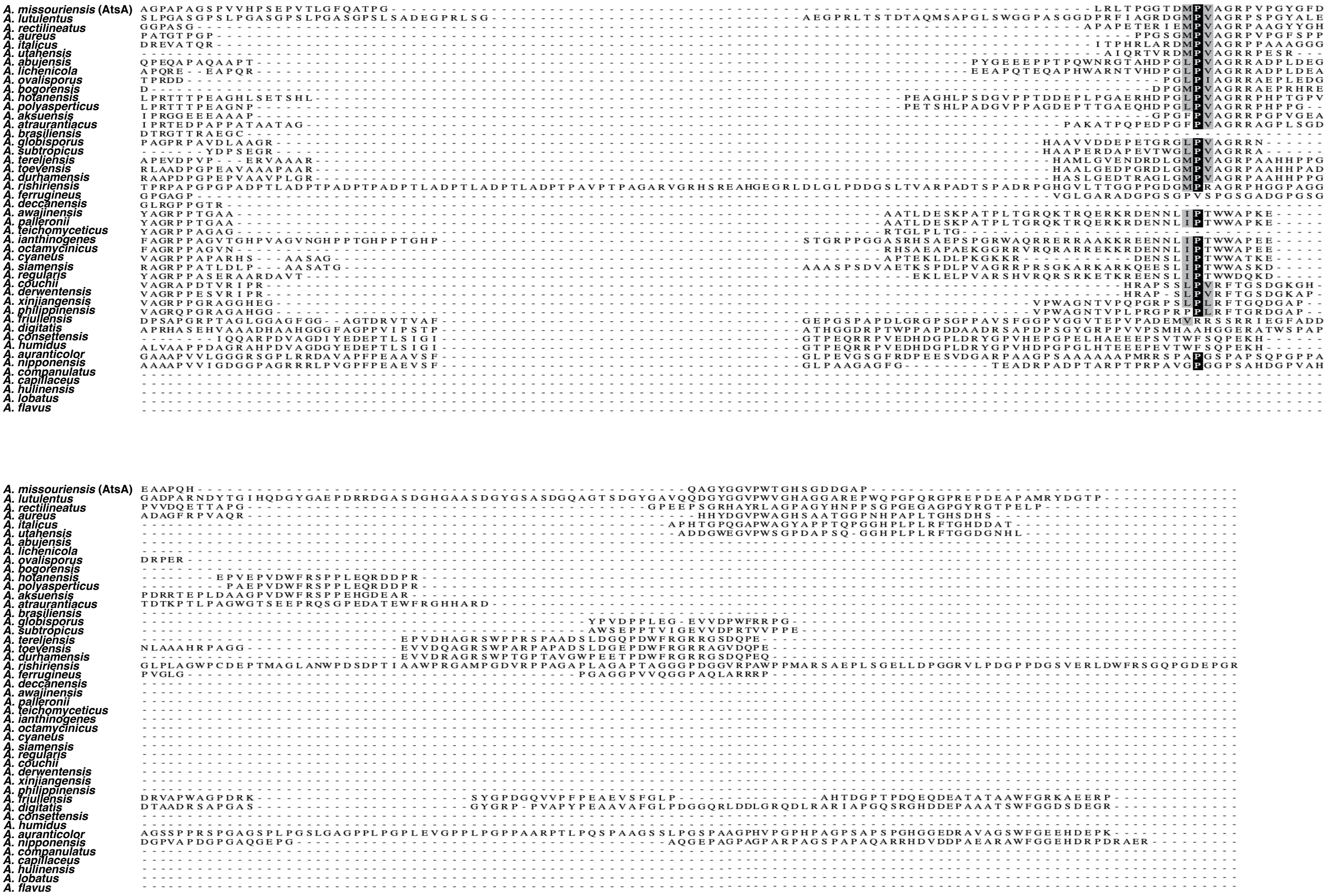

Fig. S7. Amino acid sequence alignment of AtsA and its orthologs from 46 *Actinoplanes* bacteria. The location of the acyltransferase_3 domain in AtsA is indicated by the gray rectangles.

Table S1. Primers used in this study

Primer name	Sequence (5' to 3')[a]	Restriction enzyme	Used for
AMIS_760-UF1	GCGAATTCGAGGAGTACCGGGAGCTCTA	EcoRI	Gene disruption
AMIS_760-UR1	GCTCTAGATTGATCCGGGACATCGGCGT	XbaI	Gene disruption
AMIS_760-DF1	GCTCTAGATCTAGACTCCTTCGACCACCGGATCA	XbaI	Gene disruption
AMIS_760-DR1	CCCAAGCTTGCTGCGGGAATCCGGAACCA	HindIII	Gene disruption
AMIS_11860-UF1	GCGAATTCCCCGAGTCGAAACCGGTCGA	EcoRI	Gene disruption
AMIS_11860-UR1	GCTCTAGAGGTGAGGTGGTAGACCACGA	XbaI	Gene disruption
AMIS_11860-DF1	GCTCTAGATCAGCGGGTCGTCACGGAGA	XbaI	Gene disruption
AMIS_11860-DR1	CCCAAGCTTACCGCCGTAGTTCACGCAGA	HindIII	Gene disruption
AMIS_14480-UF1	GCGAATTCCACGGCCACCTGGACCATCA	EcoRI	Gene disruption
AMIS_14480-UR1	GCTCTAGACAGCAATGGTCCGAGGACCA	XbaI	Gene disruption
AMIS_14480-DF1	GCTCTAGAGTCGACGTCTACGCCTCGAC	XbaI	Gene disruption
AMIS_14480-DR1	CCCAAGCTTACCGATCGAGACCAGGTCGA	HindIII	Gene disruption
AMIS_26070_UF	GGAATTCCGGACCGTCGACATGCATGT	EcoRI	Gene disruption
AMIS_26070_UR	GCTCTAGAAAGGCCACTGCCAGAGCACA	XbaI	Gene disruption
AMIS_26070_DF	GCTCTAGACTGATCATCACGCTCGCGTA	XbaI	Gene disruption
AMIS_26070_DR	GGCAAGCTTCTGAAGCTGGTCGATGAGCA	HindIII	Gene disruption
AMIS_30940_UF	GGAATTCGGTCCTGCTGATCGAGGAGT	EcoRI	Gene disruption
AMIS_30940_UR	GCTCTAGAGGCCATGACGGCTCGGTAGA	XbaI	Gene disruption
AMIS_30940_DF	GCTCTAGACGGCTCAAGGTGACGTTCTT	XbaI	Gene disruption
AMIS_30940_DR	GGCAAGCTTCCGGAGCTCGGACTTGTCGA	HindIII	Gene disruption
AMIS_38540_UF	GGAATTCGGCACAGAAGTTCCTGCTCA	EcoRI	Gene disruption
AMIS_38540_UR	GCTCTAGAACGCGCAGGAGATCGAAGTA	XbaI	Gene disruption
AMIS_38540_DF	GCTCTAGAGATCTCACCGCGACGGTGGT	XbaI	Gene disruption
AMIS_38540_DR	GGCAAGCTTCAGCACACCGTTGGCAGCGA	HindIII	Gene disruption
AMIS_41550_UF	GGAATTCGCGTCGATGTGACGGTGGAT	EcoRI	Gene disruption
AMIS_41550_UR	GCTCTAGAATGATTGGCGGCCAGGATCA	XbaI	Gene disruption
AMIS_41550_DF	GCTCTAGACATGCGGTGATCCCGTACGA	XbaI	Gene disruption
AMIS_41550_DR	GGCAAGCTTAGGATGGCCGGTGACCGTGA	HindIII	Gene disruption
AMIS_50540_UF	GCTCTAGAAGCGTCGTGTTCCGCGAGAT	XbaI	Gene disruption
AMIS_50540_UR	AACTGCAGGCCTCGTTCGCGAAGAACAT	PstI	Gene disruption
AMIS_50540_DF	AACTGCAGCGTGGAGCAGCCGCTGATGA	PstI	Gene disruption
AMIS_50540_DR	GGCAAGCTTATGCGCTACGTGCCGTACAA	HindIII	Gene disruption
AMIS_52390_UF	GGAATTCTCGACGATCTCCTCGACGTC	EcoRI	Gene disruption
AMIS_52390_UR	GCTCTAGACGGCCATCCGAAGTGGTGAT	XbaI	Gene disruption
AMIS_52390_DF	GCTCTAGACACGCTCGGTTTCCAGGCAA	XbaI	Gene disruption
AMIS_52390_DR	GGCAAGCTTCAGCACGATCCGCTCCTCGT	HindIII	Gene disruption
AMIS_53620_UF	GGAATTCCGATCAAGAGTCGGTGACGA	EcoRI	Gene disruption
AMIS_53620_UR	GCTCTAGAGAAGGCGCGGTAGATCTGCA	XbaI	Gene disruption
AMIS_53620_DF	GCTCTAGAGTCATCGCCTGATCGAGCGA	XbaI	Gene disruption
AMIS_53620_DR	GGCAAGCTTTCACCGACCAGGAGCGATGA	HindIII	Gene disruption
AMIS_61980_UF	GGAATTCGAAGTCCAGCTCGACGGCGT	EcoRI	Gene disruption
AMIS_61980_UR	GCTCTAGAAGGTGGTTGGCGCAGACGAT	XbaI	Gene disruption
AMIS_61980_DF	GCTCTAGAGCGCTCACCGACGAGCTGAT	XbaI	Gene disruption
AMIS_61980_DR	GGCAAGCTTATCGGTCTTCGACGGCCGGA	HindIII	Gene disruption

AMIS_62010_UF	GGAATTCCTGAGCGGACAGGAATACGT	EcoRI	Gene disruption
AMIS_62010_UR	GCTCTAGAAACAGGCGCAGACCGTCGAT	XbaI	Gene disruption
AMIS_62010_DF	GCTCTAGACCTCTGCGCCTTCATCTTCT	XbaI	Gene disruption
AMIS_62010_DR	GGCAAGCTTGATCCGGTTCAACGACACCT	HindIII	Gene disruption
AMIS_62550-UF1	GCGAATTCAGGGCGACGACCTTCCGGTA	EcoRI	Gene disruption
AMIS_62550-UR1	GCTCTAGAAGAGGACCACGAGGGTCACC	XbaI	Gene disruption
AMIS_62550-DF1	GCTCTAGACAGCCACATCAGCACCGGGT	XbaI	Gene disruption
AMIS_62550-DR1	CCCAAGCTTACGCGTGATCACCTCACCGA	HindIII	Gene disruption
AMIS_62890-UF1	GCGAATTCGCATCGACCACGGTTCCATC	EcoRI	Gene disruption
AMIS_62890-UR1	GCTCTAGATCTCCCGTAGCCGCTGTGTG	XbaI	Gene disruption
AMIS_62890-DF1	GCTCTAGAAGGACTGGCACATGCTGCAG	XbaI	Gene disruption
AMIS_62890-DR1	CCCAAGCTTGCGTACCCCGGAAAGTCAGA	HindIII	Gene disruption
AMIS_70460-UF1	GCGAATTCCGGTCGGTGGCGATCATGCA	EcoRI	Gene disruption
AMIS_70460-UR1	GCTCTAGAGGTGATCAGGAAGCCGGAGA	XbaI	Gene disruption
AMIS_70460-DF1	GCTCTAGAGACCCGAAACTCCCCTGGCA	XbaI	Gene disruption
AMIS_70460-DR1	CCCAAGCTTCCGGAGCACCGGTCGTACAA	HindIII	Gene disruption
AMIS_70880_UF	GGAATTCTTCAGCGAACGGCTCGAGAT	EcoRI	Gene disruption
AMIS_70880_UR	GCTCTAGAACGTGGTTCGGCACCAGGAT	XbaI	Gene disruption
AMIS_70880_DF	GCTCTAGAGAGATGACCGACGCGATCAT	XbaI	Gene disruption
AMIS_70880_DR	AAAACCTGCAGGCGTCACGTCCGCGATCAGAT	Sse8387I	Gene disruption
AMIS_72650_UF	GCTCTAGATTCCACGATCTGCCACCGCA	XbaI	Gene disruption
AMIS_72650_UR	AAAACCTGCAGGAGCGTGGTGACGAGCGTCGT	Sse8387I	Gene disruption
AMIS_72650_DF	AAAACCTGCAGGCGACTGGCTCTACGACTGGT	Sse8387I	Gene disruption
AMIS_72650_DR	GGCAAGCTTGTAGAGCTCGACGACGTCCT	HindIII	Gene disruption
AMIS_72750-UF1	GCGAATTCCGATCGAACCCGATGCCATC	EcoRI	Gene disruption
AMIS_72750-UR1	GCTCTAGAAAGCAGGCTGCCAGGTCGTG	XbaI	Gene disruption
AMIS_72750-DF1	GCTCTAGACCCGATCTCGATCAGCTAC	XbaI	Gene disruption
AMIS_72750-DR1	CCCAAGCTTCAGCAGCAGCAGGTCCTCCA	HindIII	Gene disruption
AMIS_73450-UF1	GCGAATTCGCCATGCCAGCTGAGCACTC	EcoRI	Gene disruption
AMIS_73450-UR1	GCTCTAGAGAGTCTTCGCCAGACCGACA	XbaI	Gene disruption
AMIS_73450-DF1	GCTCTAGAAGAAGGCGTACCCGGAGAAG	XbaI	Gene disruption
AMIS_73450-DR1	CCCAAGCTTCGGATGATCACGGATCCGGA	HindIII	Gene disruption
AMIS_75890-UF1	GCGAATTCGTCCGCGTCTTCCTTCCCTA	EcoRI	Gene disruption
AMIS_75890-UR1	GCTCTAGATCGGCAACTGGAACTCGGCA	XbaI	Gene disruption
AMIS_75890-DF1	GCTCTAGAACGACCCGCTCGTCGTCTAC	XbaI	Gene disruption
AMIS_75890-DR1	CCCAAGCTTCCGTCTTCCGGTTCGTCTAC	HindIII	Gene disruption
AMIS_77300_UF	GGAATTCTAATGCTGGTCCGAGGGCAT	EcoRI	Gene disruption
AMIS_77300_UR	GCTCTAGATAGTGGTGTGCCATGACGGA	XbaI	Gene disruption
AMIS_77300_DF	GCTCTAGAGGCGTCATGGCGATGATGCT	XbaI	Gene disruption
AMIS_77300_DR	GGCAAGCTTCAGTTCCTCGTCGGTGCGCA	HindIII	Gene disruption
atsA-SF1	GGAATTCTTGACCAACGGCCGCTGATT	EcoRI	Gene complementation
atsA-SR1	GCCAAGCTTTCAGTGAGCTTCGCTCGCTT	HindIII	Gene complementation

[a] The recognition sequences for restriction enzymes are underlined.

Table S2. Putative acyltransferase genes in the *A. missouriensis* genome

Gene ID	Length (aa)	Conserved domain[a]	Region (aa)[a]	*E* value[a]	wild-type/Δ*tcrA*[b]		wild-type/Δ*hhkA*[b]		wild-type/Δ*fliA1* Δ*fliA2* Δ*fliA3*[b]		wild-type/Δ*bldC*[c]	
					Fold change	*q* value	Fold change	*q* value	Fold change	*q* value	Fold change	*q* value
AMIS_760	485	Branched-chain α-keto acid dehydrogenase subunit E2 (acyltransferase subunit) (PRK11856)	7-482	$8.17e^{-148}$	0.89	0.02	0.98	0.72	0.81	0.04	1.15	0.08
AMIS_11360	224	Lysophospholipid acyltransferase of glycerophospholipid biosynthesis (LPLAT_AGPAT-like)	17-201	$4.42e^{-50}$	0.99	16.26	0.98	3.63	0.43	0.00	1.12	0.86
AMIS_11860	390	Acyltransferase_3	3-288	$6.96e^{-7}$	0.87	0.19	0.80	0.01	1.26	1.41	1.36	0.19
AMIS_14480	227	Lysophospholipid acyltransferase of glycerophospholipid biosynthesis (LPLAT_AGPAT-like)	14-201	$2.71e^{-56}$	0.95	1.62	1.02	4.89	0.35	0.00	1.38	0.23
AMIS_26070	406	Peptidoglycan/LPS *O*-acetylase OafA/YrhL containing acyltransferase and SGNH-hydrolase domains (OafA)	19-351	$6.11e^{-22}$	1.46	0.21	1.00	27.74	1.09	13.26	1.05	22.68
AMIS_30940	212	Lysophospholipid acyltransferase of glycerophospholipid biosynthesis (LPLAT_AGPAT-like)	34-205	$1.23e^{-37}$	0.77	0.36	0.85	0.79	0.66	0.02	1.21	1.25
AMIS_38540	455	Acyltransferase_3	27-336	$1.28e^{-17}$	0.83	0.12	1.09	0.61	0.34	0.01	1.43	0.01
AMIS_41550	254	Lysophospholipid acyltransferase of glycerophospholipid biosynthesis (LPLAT_AGPAT-like)	22-202	$1.53e^{-27}$	0.82	0.25	0.89	0.45	0.94	3.93	1.05	3.58
AMIS_50540	402	Peptidoglycan/LPS *O*-acetylase OafA/YrhL containing acyltransferase and SGNH-hydrolase domains (OafA)	1-374	$1.22e^{-21}$	1.09	0.47	1.32	0.05	1.63	1.60	0.91	0.11
AMIS_52390 (AtsA)	450	Acyltransferase_3	3-324	$6.06e^{-14}$	0.66	0.22	0.84	1.47	0.45	0.01	2.32	0.09
AMIS_53620	396	Peptidoglycan/LPS *O*-acetylase OafA/YrhL containing acyltransferase and SGNH-hydrolase domains (OafA)	1-372	$2.37e^{-12}$	0.68	0.01	1.03	6.76	0.54	0.00	1.30	0.53
AMIS_61980	220	Lysophospholipid acyltransferase of glycerophospholipid biosynthesis (LPLAT_AGPAT-like)	21-201	$2.95e^{-49}$	0.92	3.41	1.09	3.05	0.68	0.70	1.48	0.06
AMIS_62010	437	Peptidoglycan/LPS *O*-acetylase OafA/YrhL containing acyltransferase and SGNH-hydrolase domains (OafA)	15-368	$2.22e^{-26}$	1.07	0.81	1.10	0.35	0.45	0.01	1.09	0.35
AMIS_62550	744	Peptidoglycan/LPS *O*-acetylase OafA/YrhL containing acyltransferase and SGNH-hydrolase domains (OafA)	22-428	$9.77e^{-59}$	1.10	0.20	0.92	0.29	1.10	0.37	1.05	0.39
AMIS_62890	288	Lipid A biosynthesis lauroyl acyltransferase (PRK07920)	7-288	$4.82e^{-136}$	1.05	2.91	0.97	4.80	0.47	0.01	1.25	0.16
AMIS_70460	685	Peptidoglycan/LPS *O*-acetylase OafA/YrhL containing acyltransferase and SGNH-hydrolase domains (OafA)	51-344	$6.05e^{-42}$	0.75	0.00	1.10	0.50	0.29	0.00	2.68	0.02
AMIS_70880	241	Lysophospholipid acyltransferase of glycerophospholipid biosynthesis (LPLAT_AGPAT-like)	28-202	$8.78e^{-48}$	0.80	0.01	1.06	0.29	0.64	0.03	1.09	0.34
AMIS_72650	349	Lysophospholipid acyltransferase of glycerophospholipid biosynthesis (LPLAT_AGPAT-like)	104-267	$1.78e^{-14}$	1.10	0.38	1.05	1.19	1.97	0.03	0.69	0.19
AMIS_72750	300	Lysophospholipid acyltransferase of glycerophospholipid biosynthesis (LPLAT_AGPAT-like)	104-265	$1.19e^{-36}$	0.94	6.38	0.98	15.71	1.67	0.94	1.24	0.40
AMIS_73450	243	Lysophospholipid acyltransferase of glycerophospholipid biosynthesis (LPLAT_AGPAT-like)	12-197	$4.74e^{-53}$	0.88	0.35	0.99	18.36	0.44	0.01	1.04	6.41
AMIS_75890	330	Lysophospholipid acyltransferase of glycerophospholipid biosynthesis (LPLAT_AGPAT-like)	97-322	$5.17e^{-44}$	0.96	1.09	0.93	1.12	0.82	0.88	1.21	0.32
AMIS_77300	384	Peptidoglycan/LPS *O*-acetylase OafA/YrhL containing acyltransferase and SGNH-hydrolase domains (OafA)	67-375	$1.95e^{-21}$	0.73	0.03	0.94	3.60	0.70	0.10	1.06	2.38

[a] Conseved domains, regions, and *E* values in each gene product identified by the *in silico* search using the Conserved Domain Database v3.20 (https://www.ncbi.nlm.nih.gov/Structure/cdd/wrpsb.cgi) are shown. The threshold of the *E* value is 0.01.

[b] Fold changes of the average RPKM values and *q* values in the wild-type and mutant strains cultivated on HAT agar at 30°C for 6 days are shown (1, 2).

[c] The wild-type and Δ*bldC* strains were cultivated on HAT agar at 30°C for 3 days (3).

Supplemental references

1. Hashiguchi Y, Tezuka T, Mouri Y, Konishi K, Fujita A, Hirata A, Ohnishi Y. 2020. Regulation of sporangium formation, spore dormancy, and sporangium dehiscence by a hybrid sensor histidine kinase in *Actinoplanes missouriensis*: relationship with the global transcriptional regulator TcrA. *J Bacteriol* **202**:e00228-20.
2. Hashiguchi Y, Tezuka T, Ohnishi Y. 2020. Involvement of three FliA-family sigma factors in the sporangium formation, spore dormancy and sporangium dehiscence in *Actinoplanes missouriensis*. *Mol Microbiol* **113**:1170-1188.
3. Tezuka T, Nitta S, Ohnishi Y. 2022. Involvement of BldC in the formation of physiologically mature sporangium in *Actinoplanes missouriensis*. *J Bacteriol* **204**:e0018922.
